# Dataset demonstrating physical properties of recycled wind turbine blade composites

**DOI:** 10.1016/j.dib.2018.08.123

**Published:** 2018-08-31

**Authors:** Seyed Hossein Mamanpush, Hui Li, Karl Englund, Azadeh Tavousi Tabatabaei

**Affiliations:** Composite Materials and Engineering Center, Washington State University, Pullman, WA, 99163, USA

## Abstract

Wind turbine blades that face end-of-life recycled mechanically. The recycled material was first comminuted via a hammer-mill through a range of varying screen sizes, resinated and compressed to a final thickness to manufacture second generation composites fabricated using recycled wind turbine material and a polyurethane adhesive. Physical properties (water sorption (WA), Thickness swelling (TS)) dataset of composites made from recycled wind turbine blades presented. Dataset also presented the influence of resin level, moisture content, mill screen size and density on the physical properties of composites.

**Specifications Table**

**Table:** 

Subject area	Composite material
More specific subject area	Recycling thermoset composite material
Type of data	Table
How data was acquired	Physical properties are obtained by experimental method using ASTM 1037-12
Data format	Analyzed
Experimental factors	
Experimental features	
Data source location	Composites Materials and Engineering Center at Washington State University
Data accessibility	
Related research article	Seyed Hossein Mamanpush, et.al., Recycled wind turbine blades as a feedstock for second generation composites [1]

**Value of the data**

• Thickness swelling and water sorption of recycled wind turbine blade composites presented that give the researchers clear vision about their physical properties.
• Researchers could be referred to this dataset to design and analyze different experiments on recycled win turbine blades.
• Presented comparison between the recycled wind turbine blade composites and wood-base composites shows the potential utilization of this recycled material in different fields.

## 1 Data

For obtaining physical properties of composites fabricated using recycled wind turbine blade materials, water sorption and thickness swelling were performed based on ASTM D1037-12. Presented dataset include influence of resin level (MDI(%)), moisture content(MC(%)), mill screen size (MSS(mm)) and density on the physical properties of second generation composites.

## 2 Experimental design, materials, and methods

### 2.1 Materials

Recycled wind turbine blade (rWTB) material supplied by Global Fiberglass Solutions at an incoming MC of 1.25%. A polymeric methyl-diisocyanate (Rubinate 1840) (pMDI) resin was kindly supplied by Huntsman and was used as the binder for the second generation panel product. The rWTB material was then hammer-milled through 12.7, 6.35, 3.18, and 1.59 mm screen size, respectively.

### 2.2 Manufacturing of rWTB composites

The various size fractions of the rWTB materials were sprayed with resin and water (to obtain the targeted MC) within a drum blender. The blended rWTB was then hand-formed and hot pressed to a size of 355.6×355.6mm composites panels (duplicate) with a thickness of 7.62 mm.

### 2.3 Physical properties

#### 2.3.1 Thickness swelling

Results of thickness swelling are given in Table 1. The lowest amount of thickness swelling after 2h of immersion was found for the composite with 3.18 mm MSS and density of 1.12 g/cm^3^ (MDI = 6 % and MC = 5%) and is equal to 0.11%, also after 24 h of immersion the rWTB composites with 1.59 mm MSS and density of 1.04 g/cm^3^ has the minimum amount of swelling equal to 0.71%. Comparing the results of these two rWTB composites with the wood-base particleboard [2] shows that thickness swelling of these two composites are 0.88 % and 2.63% of wood-base particleboard respectively. With resin levels, we see that after 24h of immersion, by increasing the amount of MDI from 3% to 10% thickness swelling reduced from 3.7 % to 1.9 %, Evaluating the influence of MC on the thickness swelling shows that after 24 h of immersion there is no considerable difference between the thickness swelling of rWTB composites with MC of 3% and 5%, but by increasing the MC to 8% the thickness swelling increased slightly in the 2 h immersion data.

**Table 1:** water resistance properties of wood-based composite bond with 4% PMDI [2].

	Water sorption (%)	Thickness swelling (%)
2 h	16.99	12.5
24 h	48.22	26.95

Investigating the influence of density on the thickness swelling of rWTB composites showed no consistent trend. After 2 h of immersion rWTB composites with a density of 1.04 g/cm^3^ swelled more compared to the rWTB composites with densities of 1.12 and 0.88 g/cm^3^. but after 24 h of immersion this composite swell less. The influences of MSS showed that after 24 h of immersion, thickness swelling reduced when the smallest MSS material was used. Thickness swelling is 1.9 % for rWTB composites with 12.7 mm MSS, but for the rWTB composites with 1.59 mm MSS thickness swelling is reduced to 0.71%.

#### 2.3.2 Water sorption

The water sorption dataset are presented in Table 2. The minimum amount of water sorption after 2 h of immersion is 1.18% and is for the rWTB composites with MDI = 6%, MC = 5% and 6.35 mm MSS, after 24 h of immersion the same composite absorbed minimum amount of water equal to 4.51%. These results indicate a substantial 90% or greater reduction in water sorption properties in the 2 h timeframe.

**Table 2:** Physical properties of rWTB composites considering (a) MDI (%) (b) MC (%) (c) MSS and (d) density.

MSS (mm)	MDI(%)	MC(%)	Density (g/cm3)	WA-2 H	WA-24 H	TS-2 H	TS-24 H
12.7	10	5	1.04	2.098977118	5.203098208	0.643365034	1.677041331
12.7	10	5	1.04	1.525299713	4.947374346	1.330716882	2.080649328
12.7	3	5	1.04	2.647358298	7.913791153	1.425488542	3.359680772
12.7	3	5	1.04	2.830188679	8.573632372	2.49659785	4.012985213
12.7	6	3	1.04	1.378093821	5.280855521	1.488934598	2.081399343
12.7	6	3	1.04	1.253721987	4.450713054	0.647927625	1.795139707
12.7	6	8	1.04	1.505308192	4.743041251	1.862337893	2.596947575
12.7	6	8	1.04	1.720063747	4.86893444	1.473739242	2.204667897
12.7	6	5	1.04	1.888579387	5.370473538	0.930039413	2.087691452
12.7	6	5	1.04	1.427559527	4.931073939	0.835244419	1.777356143
6.35	6	5	1.04	1.131071191	4.235972499	0.534541081	1.488468767
6.35	6	5	1.04	1.234989471	4.791986796	1.372971913	2.216528408
3.18	6	5	1.04	2.325292216	6.105446406	0.922587852	1.191693657
3.18	6	5	1.04	1.158084102	4.660445244	0.822831143	1.441065826
3.18	6	5	1.12	1.73571054	5.794441531	0.15625123	1.09375123
3.18	6	5	1.12	1.947097722	5.883279102	0.152439024	0.914634146
3.18	6	5	0.88	1.458655332	5.443392581	0.152439024	2.134146341
3.18	6	5	0.88	1.867003459	6.353302949	0.148809524	1.041666667
1.59	6	5	1.04	2.294432798	6.766829723	0.24653268	0.635930048
1.59	6	5	1.04	2.467713575	7.585036076	0.158730159	0.952380952

Considering the influences of MDI on the water sorption of rWTB composites show that after 24h of immersion by increasing the MDI the amount of sorbed water reduced, by increasing the MDI level from 3% to 6% the amount of water sorption reduced however, little improvement in water resistance was not observed when the resin level was increased to 10%. The role of MC and density had little influence on the water sorption properties.
